# Preceding Viral Infections Do Not Imprint Long-Term Changes in Regulatory T Cell Function

**DOI:** 10.1038/s41598-020-65212-9

**Published:** 2020-05-20

**Authors:** Felix Rost, Katharina Lambert, Nikolas Rakebrandt, Nicole Joller

**Affiliations:** 1 University of Zurich, Institute of Experimental Immunology, Zurich, 8057 Switzerland; 2 Present Address: Translational Research Program, Benaroya Research Institute, Seattle, WA 98101 USA; 3 Present Address: F. Hoffmann-La Roche, Basel, 4070 Switzerland

**Keywords:** Immunology, Infection

## Abstract

Regulatory T cells (T_regs_) maintain peripheral self-tolerance and limit immune mediated pathology. Like effector T cells, T_regs_ can specialize in T_H_1-dominated immune responses and co-express T-bet together with Foxp3. This allows for expression of CXCR3 and efficient homing to sites of T_H_1 responses. However, whether such functional specialization is paralleled by memory generation among T_regs_ is unknown. In this study, we investigated the ability of polyclonal T_regs_ to form functional memory in response to viral infection. Using adoptive transfer models to compare infection-experienced T_regs_ generated upon acute Lymphocytic Choriomeningitis Virus (LCMV) WE and Vaccinia Virus (VV) infections with naive T_regs_, we observed no differences in their phenotype or their *in vivo* maintenance. When comparing functional properties of infection-experienced and naive T_regs_, we found no differences in *in vitro* suppressive capacity nor in their ability to limit the effector response upon homologous, systemic or local re-challenge *in vivo*. Our results suggest that no functional T_reg_ memory is generated in the context of systemic LCMV or VV infection, but we cannot rule out the possibility that the generation of T_reg_ memory may be possible in other contexts.

## Introduction

Regulatory T cells (T_regs_) restrain autoreactive effector T cells that have escaped negative selection in the thymus to maintain self-tolerance, and negatively regulate immune responses. During infections, T_reg_-mediated immune regulation requires tight coordination to allow for efficient pathogen clearance by effector cells while minimizing immune-mediated damage to host cells and tissues^1^. In the context of a primary immune response pathogen-specific CD4+ effector T cells can terminally differentiate into T helper 1 (T_H_1), T_H_2, T_H_17 or T_FH_ cells^2^. Once a pathogenic threat has been neutralized, activated effector T cells undergo contraction after which only a small number of pathogen-specific memory T cells remain. These long-lived memory cells can, upon pathogen re-encounter, respond with an enhanced kinetic and magnitude and render secondary immune responses more effective^3^.

Primary viral infections predominantly induce a T_H_1 effector differentiation program that is driven by the master transcription factor T-bet, which regulates expression of the T_H_1-specific chemokine receptor CXCR3^4^. In parallel to effector T cells, a fraction of the Foxp3+ T_reg_ population can mirror this specialization program by co-expressing T-bet and Foxp3 and up-regulating CXCR3. This process is dynamically regulated, specific and essential for the effective control of T_H_1 polarized immune responses^5,6^ and enables T_regs_ to accumulate in tissues of T_H_1 mediated inflammation^7^. CXCR3+ T_regs_ were found to be potent suppressors of T_H_1 cells and upregulate markers associated with suppressive function such as LAG-3 or CD85k^8^. Several groups, including our own, have reported an induction of CXCR3+ T_regs_ during the acute phase of a T_H_1 response^5,6,8^. However, whether, in line with memory generation in effector T cells, this functional specialization also leads to long-term changes in T_regs_ and the generation of T_reg_ memory, is still unclear.

Several important aspects separate effector from memory T cells. These include long-term persistence in the absence of cognate antigen and enhanced response kinetics and/or function upon homologous re-challenge^1^. However, applying these criteria to establish if T_reg_ memory can be generated is problematic. Firstly, classical memory markers of effector T cells such as CD44 are already expressed by a considerable fraction of T_regs_ under homeostatic conditions^9^, and indeed until today no reliable markers of memory T_regs_ have been identified^1^. However, because T-bet and CXCR3 were reported to be induced and stably expressed by T_regs_ during and after acute T_H_1 responses^5,6^ both may represent useful markers to identify cells with the capacity to generate memory. Secondly, detecting persistence of non-self-specific memory T_regs_ in the absence of their cognate antigen *in vivo* is complicated by the fact that the T_reg_ TCR repertoire is presumed to be largely skewed towards recognizing self-antigen^10^ and thus pathogen-specific T_regs_ may be low in number for some pathogens, or completely absent for others. This observation does, however, not preclude the existence of pathogen-specific T_regs_ *in vivo* and, indeed, several research groups have successfully identified pathogen-specific T_regs_ by tetramer staining in mice^11–14^. Consequently, murine *in vivo* models of acute, transient infection may allow to examine pathogen-specific memory formation by characterizing the responses and *in vivo* maintenance of an inflammation-experienced, polyclonal T_reg_ population in general and of T-bet+ or CXCR3+ T_regs_ in particular under physiologic conditions and in the absence of cognate antigen. In accordance with this, several research groups have used TCR-transgenic T_regs_ to address memory formation in models of viral infection *in vivo*. Adoptively transferred T_regs_ specific for influenza A virus (IAV) hemagglutinin (HA) expanded following an acute infection with a recombinant virus that encoded HA, then underwent contraction and upon homologous re-challenge expanded more rapidly and significantly reduced the recall expansion of effector T cells^15^. Similar observations including T_reg_ expansion, contraction and accelerated re-expansion during homologous viral re-challenge were made by another group that addressed the responses of TCR-transgenic T_regs_ specific for the IAV nucleoprotein^16^. However, whether these findings can also be applied to the endogenous, polyclonal T_reg_ population is still unclear.

In this study, we compared the phenotypic and functional properties of polyclonal T_regs_ from naive and infection-experienced mice using two models of homologous viral re-challenge. We show that infection-experienced, polyclonal T_regs_ do not exhibit superior maintenance in the absence of an immunologic challenge and do not show marked differences in their phenotype. Furthermore, naive and infection-experienced T_regs_ did not differ in their suppressive capability and do not alter systemic or localized anti-viral effector T cell responses upon homologous systemic or tissue localized re-challenge *in vivo*. In summary, we did not observe T_reg_ memory responses within the endogenous polyclonal T_reg_ pool.

## Results

### *In vitro* suppressive capacity of T_regs_ from infection-experienced and naive mice is comparable

**Figure 1:**
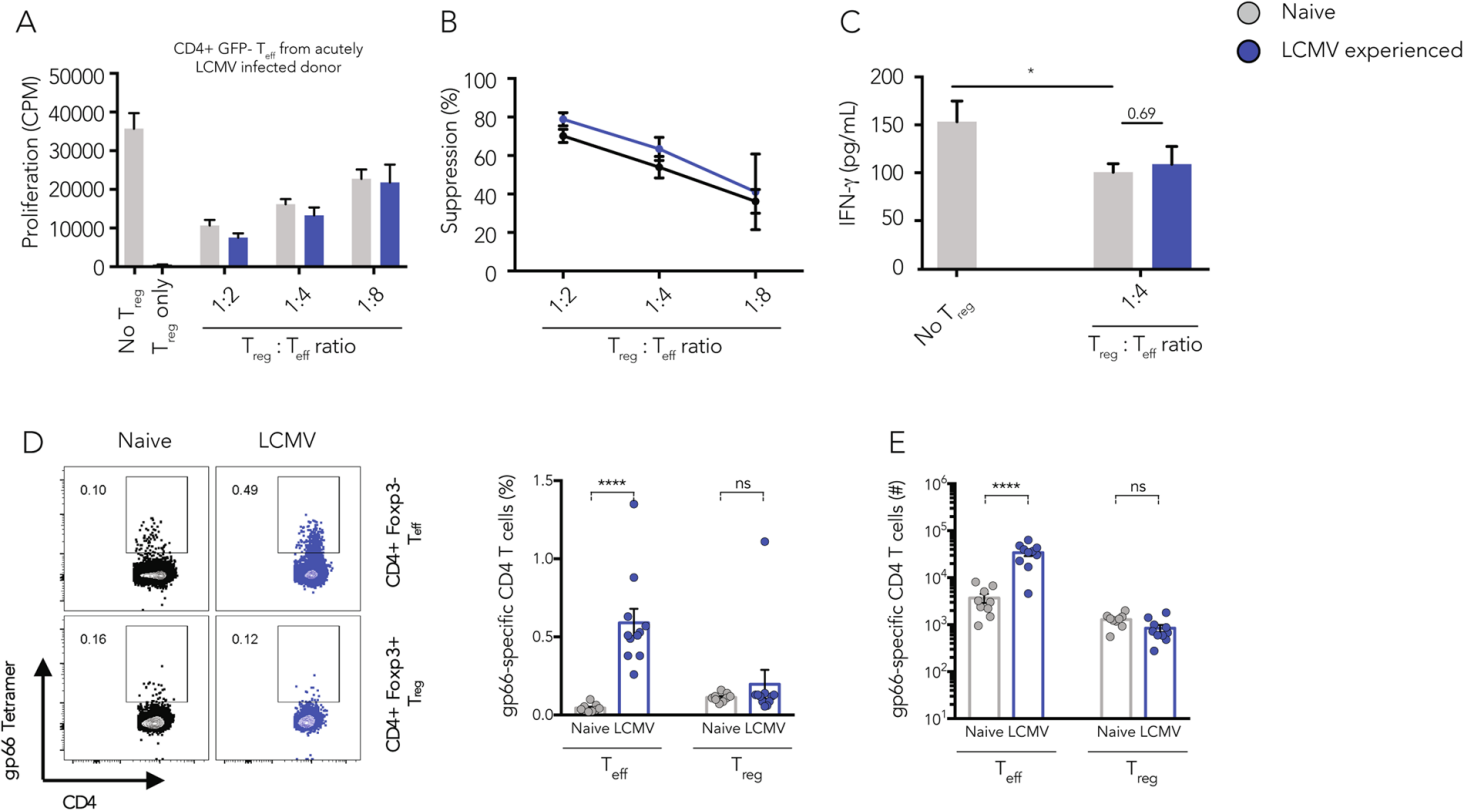
Infection-experienced T_regs_ show trend for higher *in vitro* suppressive capacity. CD4+GFP+ T_regs_ were sorted from naive or LCMV-experienced *Foxp3*-GFP reporter mice and co-cultured with CD4+GFP- effector cells isolated from acutely LCMV infected donor mice in the presence of soluble anti-CD3 (1 µg/ml) and irradiated splenic APCs isolated from naive mice for 2 days, then [^3^H]thymidine was added for 18–22 h and [^3^H]thymidine incorporation was quantified to determine proliferation. Target cell proliferation (**A**) and calculated T_reg_-mediated suppression (**B**) are shown. (**C**) The concentration of IFN-γ in the suppression assay supernatants harvested after 48 h of co-culture was determined by cytometric bead assay. (Mean ± SD; biological replicates: no T_regs_, naive T_regs_, memory T_regs_ n = 6; 2 independent experiments) (one way ANOVA and multiple comparisons test, *p < 0.05, **p < 0.01, ***p < 0.001). (**D**) Splenic T cells from naive or LCMV-experienced mice (>30 days post infection) were stained using gp66 tetramers to detect LCMV-specific CD4+Foxp3+ T_regs_ or CD4+Foxp3- effector T cells. Frequencies and absolute numbers are shown. (Mean ± SD; biological replicates: naive n = 8, LCMV-experienced n = 9; 3 independent experiments) (t Test, ****p < 0.0001).

Expression of the transcription factor T-bet and the downstream chemokine receptor CXCR3 by T_regs_ in viral infections was shown to be dynamically regulated^8^ and essential for the control of T_H_1 polarized immune responses^5,6^. However, despite clear evidence that the expression of transcription factors such as T-bet^5^, Rbpj^17^, IRF4^18^, or STAT3^19^ by T_regs_ is required to regulate T_H_1, T_H_2 or T_H_17 immune responses, it is still unclear if such phenotypic polarization is also paralleled by memory formation. It was shown that an acute LCMV infection induces the expression of T-bet and CXCR3 as well as T_reg_ effector molecules such as LAG-3, TIM-3, and CD85k at the peak of the infection^8^. However, 30–60 days after primary infection with LCMV or VV the absolute T_reg_ numbers in infection-experienced or naive mice were comparable across lymphoid and non-lymphoid organs (Supplementary Fig. 1A,B). Similarly, we observed no differences in the MFI of T-bet and the frequency of CXCR3+ T_regs_ (Supplementary Fig. 1C,D), nor in the frequency of neuropilin-1- peripherally induced pT_regs_ (Supplementary Fig. 1E,F). Still, we surmised that the infection-experienced T_reg_ pool may be enriched for memory T_regs_ of enhanced suppressive capacity that may not be marked by enhanced expression of CXCR3 and thus not be readily detectable within the total T_reg_ population. Additionally, even though CXCR3+ T_regs_ were isolated at similar frequencies from naive or infection-experienced mice (Supplementary Fig. 2A) their suppressive capacity may still be different because only T_regs_ from infection-experienced mice had been exposed to a potent inflammatory environment. Thus, we wanted to determine whether there might be differences in the suppressive capacity of polyclonal infection-experienced or naive T_regs._ To address this question, we compared their ability to suppress T_H_1 effector cells in an *in vitro* suppression assay. To generate infection-experienced T_regs_, *Foxp3*-GFP reporter mice were acutely infected with LCMV to induce a potent T_H_1 polarized immune response. CD4+ Foxp3-GFP+ T_regs_ were sorted from LCMV-experienced (day 30) or naive *Foxp3-*GFP reporter mice and co-cultured with anti-CD3 stimulated CD4+GFP− effector T cells from acutely LCMV infected mice in an *in vitro* suppression assay. We observed a pronounced and similar suppression of proliferation of CD4+GFP− effector T cells from LCMV infected mice by both naive or LCMV-experienced T_regs_ (Fig. 1A,B). The quantification of the cytokine IFN-γ in the supernatants of the suppression assays confirmed the equivalent suppressive capacity of both T_reg_ populations (Fig. 1C). We also observed similar suppression of proliferation of CD4+GFP− effector T cells isolated from naive donor mice by T_regs_ from naive or LCMV-experienced mice (Supplementary Fig. 2B,C). Next, we wanted to address whether LCMV-experienced T_regs_ might show superior LCMV-specific suppression. To this end, we tested the ability of naive and LCMV-experienced T_regs_ to suppress antigen-specific stimulation of LCMV gp61-specific SMARTA effector T cells in an *in vitro* suppression assay. We found that neither naive nor LCMV-experienced T_regs_ were able to suppress SMARTA cell proliferation despite the fact that both T_reg_ populations were functional when stimulated with anti-CD3 (Supplementary Fig. 2D). To determine whether LCMV gp66-specific T_regs_ are at all present in these T_reg_ populations, we next performed a gp66 tetramer staining. We found that splenic CD4+ Foxp3+ T_regs_ and Foxp3− effector T cells of naive mice indeed harbored LCMV gp66-specific cells at similar frequencies (Fig. 1D). However, while gp66-specific CD4+ effector T cells were detectable at higher numbers 30 days after acute LCMV infection, the absolute numbers of gp66-specific T_regs_ remained unchanged in LCMV-experienced mice, suggesting that these cells, while detectable, did not expand *in vivo* (Fig. 1E).

### T_regs_ from infection-experienced and naive mice are maintained equally well *in vivo*

Assessing the suppressive capacity of T_regs_ in an *in vitro* suppression assay is a useful, yet biologically constrained means to compare T_reg_ function. We hypothesized, that potential differences between T_regs_ from naive and infection-experienced mice might emerge *in vivo* because *in vitro* suppression assays do not allow to capture all possible suppressive mechanisms that can be employed by T_regs_ such as e.g. the impairment of effector T cell recruitment to inflammatory sites^20^. To obtain a more comprehensive overview of the functionality and relevance of potential memory formation, we next studied T_regs_ in *in vivo* models of infection.

**Figure 2:**
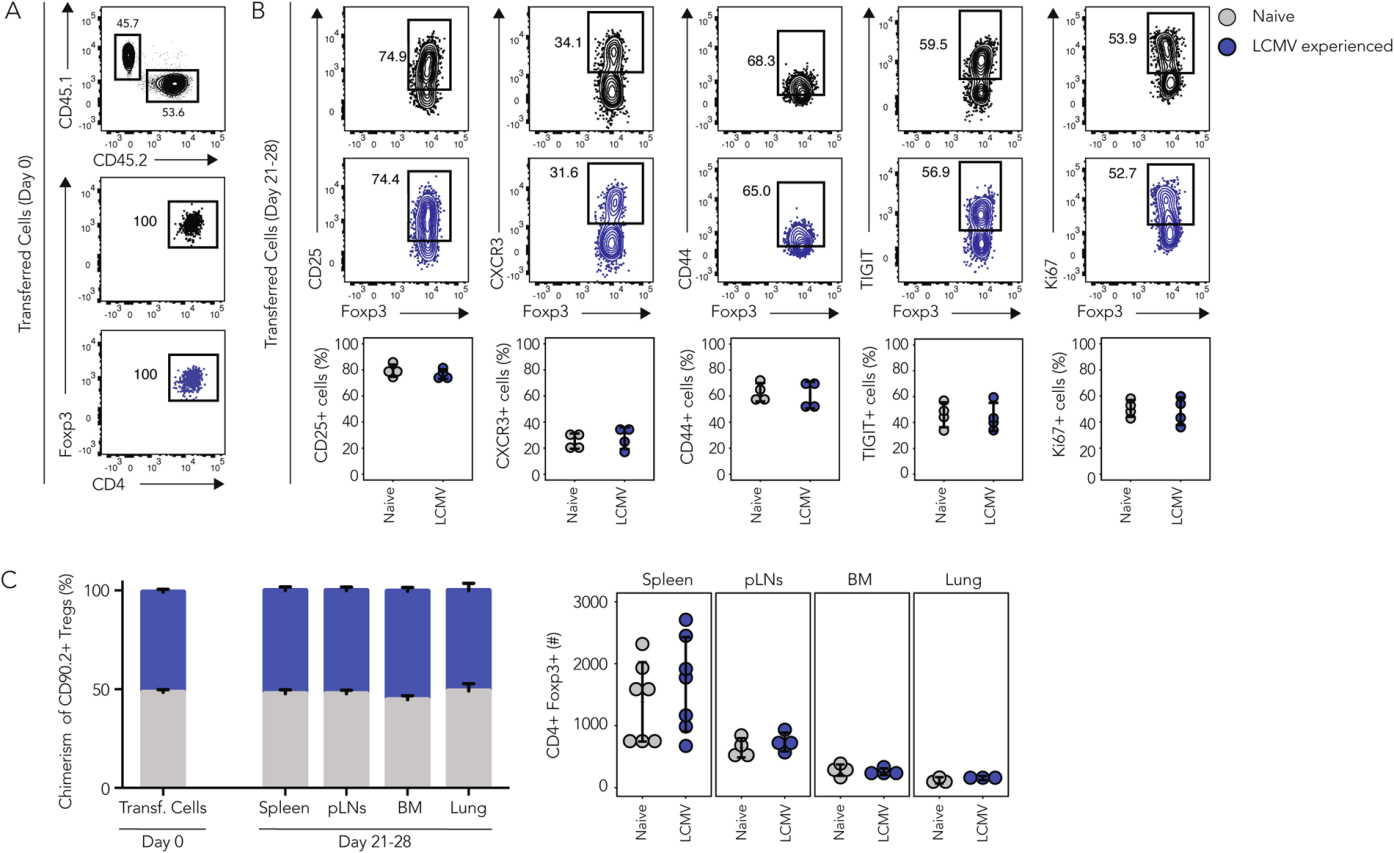
Infection-experienced and naïve T_regs_ are equally well maintained *in vivo*. CD4+GFP+ T_regs_ were sorted from naive (CD45.1) or LCMV-experienced (CD45.2) *Foxp3*-GFP reporter mice and 500’000 of each population were adoptively co-transferred i.v. into naive CD90.1+ recipient mice. Organs from T_reg_ recipients were harvested and analyzed by flow cytometry 21–28 days after transfer. (**A**) Characterization of T_regs_ from naive and LCMV-experienced mice before transfer. Input ratio (top) and purity (bottom) are shown in representative plots. (**B**) On day 21–28 post transfer, expression of CD25, CXCR3, CD44, TIGIT and Ki67 was determined in naive or LCMV-experienced CD4+Foxp3+ T_regs_ isolated from the spleens of recipient mice. Representative plots (top) and summary plots (bottom) are depicted. (**C**) Chimerism (left) and absolute numbers (right) of CD90.2^+^ T_regs_ recovered from the indicated organs on day 21 to 28 post transfer. (**B,C**) Cumulative data (Mean ± SD; biological replicates: Spleen n = 7, pLNs n = 4, BM n = 4, Lung n = 3; 4 independent experiments) (t Test, *p < 0.05, **p < 0.01, ***p < 0.001).

One defining feature of T cell memory is the stable maintenance of antigen-specific memory T cells long after the cognate antigen has been cleared^1^. We thus wondered if a polyclonal T_reg_ pool isolated from infection-experienced mice, and thus potentially enriched for memory T_regs_, would show signs of prolonged *in vivo* maintenance in the absence of an immune challenge. To test this, we performed adoptive co-transfers of congenically marked polyclonal CD4+GFP+ T_regs_ isolated from naive (CD45.1+) or LCMV-experienced (CD45.2+) *Foxp3*-GFP reporter mice at a 1:1 ratio into naive CD90.1+ recipient mice and the purity of the transferred cell population was confirmed by Foxp3 staining (Fig. 2A). The recipient mice were then sacrificed 21 to 28 days after transfer to compare the phenotype and absolute numbers of the CD45.1+ or CD45.2+ recovered T_regs_. T_regs_ recovered from the spleen of recipient mice showed similar frequencies of cells expressing the IL-2 receptor alpha chain (CD25), the chemokine receptor CXCR3, the activation marker CD44, the co-inhibitory receptor TIGIT, and the transcription factor Ki67, which marks cells that actively undergo cell cycling (Fig. 2B). Thus, there appeared to be no selective, preferential *in vivo* maintenance or enrichment of CXCR3+ LCMV-experienced compared to naive T_regs_. We also found that the 1:1 ratio of transferred naive and LCMV- experienced T_regs_ was maintained in spleen, pooled peripheral lymph nodes, bone marrow and lung of the recipient mice and we recovered similar absolute numbers of both populations in all organs tested (Fig. 2C). This suggested that the *in vivo* maintenance of naive or LCMV-experienced T_regs_ is similar across primary and secondary lymphoid organs as well as peripheral tissues such as the lung and there seemed to be no preferential homing of LCMV-experienced T_regs_ to any particular site. Overall, we did not observe any differences in the phenotype and *in vivo* maintenance of polyclonal T_regs_ isolated from naive or LCMV-experienced donor mice.

### T_reg_ cell phenotype and function in systemic viral re-challenges are comparable

**Figure 3:**
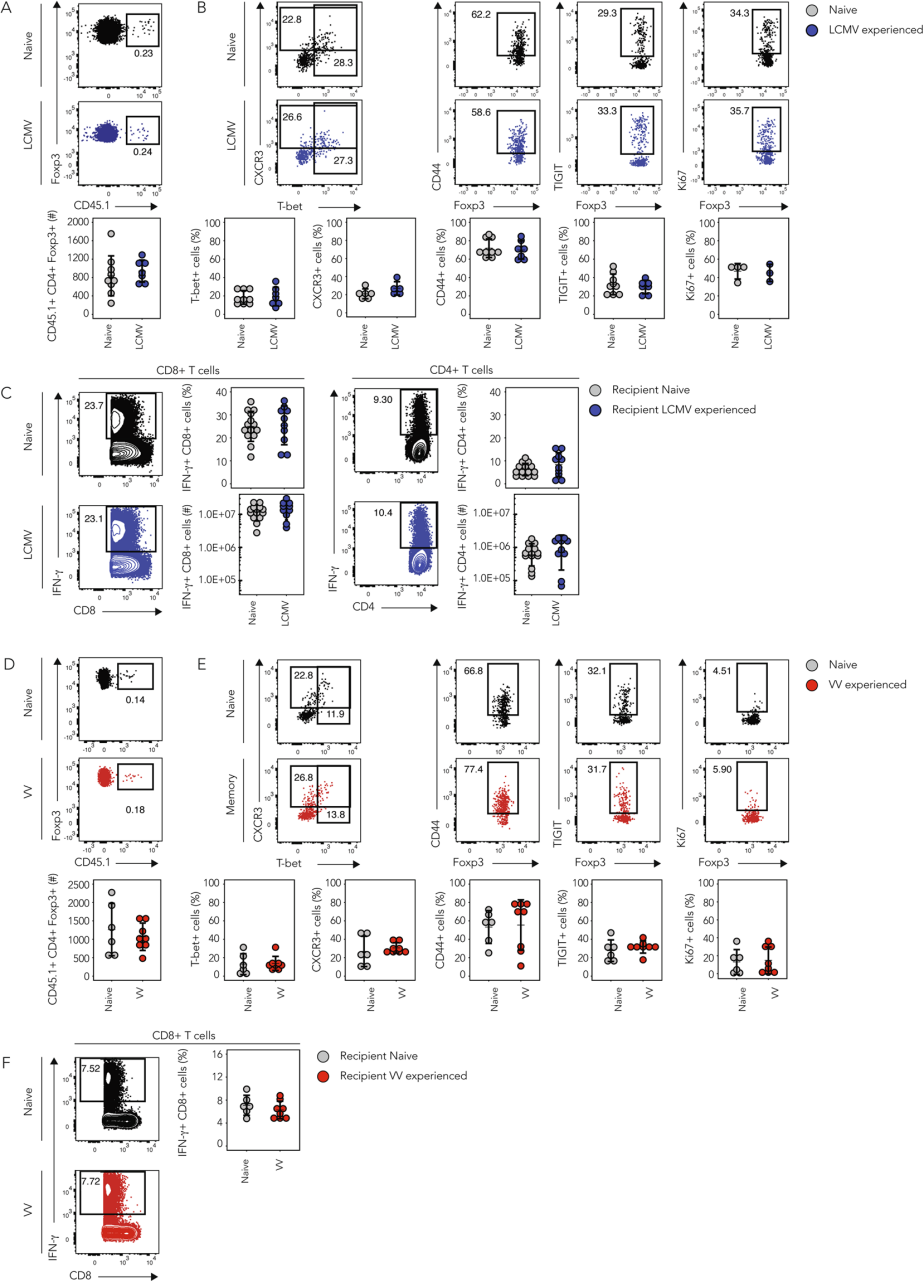
Naive and infection-experienced T_regs_ have comparable phenotypes in systemic, homologous challenges. CD4+GFP+ T_regs_ were sorted from naive, LCMV- (**A–C**) or VV- (**D–F**) experienced CD45.1 + *Foxp3*-GFP reporter mice >30 days after the primary infection and 500’000 of each population were adoptively transferred into separate groups of CD45.2+ recipient mice followed by acute LCMV (**A–C**) or VV (**D–F**) infection. (**A–C**) T_reg_ recipients were sacrificed 10 days post LCMV infection and the transferred T_regs_ characterized in the spleen. (**A**) Frequencies of transferred naive or LCMV-experienced T_regs_ within the total T_reg_ population (top) and absolute numbers of recovered, transferred T_regs_ (bottom). (**B**) Naive or LCMV-experienced transferred T_regs_ were phenotypically characterized by flow cytometry. Representative plots (top) and cumulative data (bottom) depicting frequencies of marker positive cells among the transferred T_regs_. (**C**) Splenocytes of recipient mice were re-stimulated with LCMV peptides (gp33 and gp61) for 4 h followed by intracellular cytokine staining for IFN-γ. (Mean ± SD; biological replicates: naive n = 4–14, LCMV-experienced n = 3–10; 3 independent experiments). (**D–F**) T_reg_ recipients were sacrificed 7 days post VV infection and the transferred T_regs_ characterized in the spleen (**D**). Frequencies of transferred naive or VV-experienced T_regs_ within the total T_reg_ population (top) and absolute numbers of recovered, transferred T_regs_ (bottom). (**E**) Naive or VV-experienced transferred T_regs_ were phenotypically characterized by flow cytometry. Representative plots (top) and cumulative data (bottom) depicting frequencies of marker positive cells among the transferred T_regs_. (**F**) Splenocytes of recipient mice were re-stimulated with VV peptide for 4 h followed by intracellular cytokine staining for IFN-γ. (Mean ± SD; biological replicates: naive n = 6, VV-experienced n = 8; 2 independent experiments) (t Test, *p < 0.05, **p < 0.01, ***p < 0.001).

Next, we compared the *in vivo* responses of naive or infection-experienced T_regs_ in homologous viral re-challenges and quantified their effect on the endogenous anti-viral effector T cell response. We found that adoptive transfer of 5 × 10^5^ polyclonal T_regs_ is sufficient to dampen the endogenous effector T cell response (Supplementary Fig. 3) and thus used this as a basis to assess the suppressive function of naive vs. infection-experienced T_regs_ *in vivo*. To this end, we isolated bulk T_regs_ from naive or LCMV-experienced *Foxp3*-GFP reporter mice >30 days after the primary infection and adoptively transferred 5 × 10^5^ T_regs_ into separate groups of naive, congenically marked recipient mice. 1 day after the adoptive transfer, recipient mice were challenged with LCMV and 10 days later the spleens were harvested and the phenotype of the transferred T_regs_ and the endogenous effector T cell responses were compared. We already showed earlier that the phenotype of naive or LCMV-experienced T_regs_ was comparable at the day of transfer (Supplementary Fig. 2A). The absolute numbers of naive or LCMV-experienced T_regs_ recovered from the spleen of recipient mice on day 10 after systemic viral re-challenge did not differ (Fig. 3A). This indicated that T_regs_ isolated from LCMV-experienced mice did not show signs of more pronounced expansion or superior maintenance upon homologous LCMV re-challenge *in vivo*. Furthermore, we did not observe differences in the frequency of T-bet+ or CXCR3+ cells among naive or LCMV-experienced T_regs_, indicating that the homologous re-challenge did not induce a preferential enrichment of T_H_1 suppressing T_regs_ among the LCMV-experienced T_reg_ population (Fig. 3B). Also, the expression of the memory and activation marker CD44, as well as the co-inhibitory receptor TIGIT did not differ. The frequency of Ki67+ cells was also similar and suggested no differences in cell cycling between T_regs_ isolated from naive or LCMV-experienced mice.

To determine whether T_regs_ from infection-experienced or naive mice differed in their ability to suppress effector T cells *in vivo*, we compared the endogenous effector T cell response after LCMV-specific *ex vivo* peptide re-stimulation. Again, we did not observe any differences in the frequencies or absolute numbers of splenic IFN-γ+ CD8+ or CD4+ effector T cells in recipients of naive or LCMV-experienced T_regs_ (Fig. 3C). Similarly, no differences were detected in the frequencies or absolute numbers of TNF-α+ CD8+ or CD4+ effector T cells (Supplementary Fig. 4A). Also, re-stimulation of total splenocytes using PMA/Ionomycin revealed no differences in the frequency or absolute number of IFN-γ+ CD8+ or CD4+ effector T cells (Supplementary Fig. 4B).

To exclude the possibility that the potent anti-viral immune response elicited by LCMV^21^ could mask potentially small differences in the T_reg_ phenotype or their impact on an ongoing anti-viral immune response, we performed an analogous second set of experiments using Vaccinia Virus (VV) instead of LCMV. As for LCMV, the phenotype of T_regs_ isolated from naive or VV-experienced mice was similar (Supplementary Fig. 1B,D,F and Supplementary Fig. 2A) and we adoptively transferred these bulk populations into separate groups of congenically marked recipient mice and infected them with VV one day after the transfer. 7 days after the systemic viral re-challenge the spleens of the recipient mice were harvested for analysis. As for LCMV, we did not observe differences in the absolute numbers of naive or VV- experienced T_regs_ recovered from the spleen (Fig. 3D). Flow cytometric analysis did also not show any differences in the frequency of T-bet+ or CXCR3+, CD44+, TIGIT + or Ki67+ cells among the transferred T_regs_ (Fig. 3E). Furthermore, no differences in the frequency of IFN-γ+ or TNF-α+ CD8+ effector T cells were observed after VV-specific re-stimulation (Fig. 3F, Supplementary Fig. 4C) or of IFN-γ+ CD8+ or CD4+ effector T cells after re-stimulation of total splenocytes using PMA/Ionomycin (Supplementary Fig. 4D).

Overall, neither LCMV- nor VV-experienced T_regs_ showed enhanced expression of T-bet or CXCR3, increased activation, or cell cycling. In line with this, the absolute numbers of naive or infection-experienced T_regs_ that we recovered from the spleen of recipient mice did not differ. We also did not observe differences in the magnitude of the endogenous anti-viral CD8+ or CD4+ effector T cell response in the presence of infection-experienced compared to naive T_regs_. This suggested that in our model system of adoptive T_reg_ transfer the presence of infection-experienced T_regs_ did not alter the course of the anti-viral immune response nor did the T_regs_ differ in their phenotype for the markers that we investigated.

### T_reg_ cell responses in localized viral re-challenges are comparable

We found that in systemic homologous re-challenges the phenotype and the capacity of naive or infection-experienced T_regs_ to regulate effector T cell responses was comparable. Nevertheless, as virus-specific memory T_regs_ were shown to be able to expand *in vivo*^15,16,22^, and because expression of T-bet has been reported to be stable over time^5,6^ we hypothesized that infection-experienced T_regs_ might, upon viral re-challenge, show faster recruitment to the site of a localized viral infection compared to naive T_regs_. This is because T-bet-dependent expression of CXCR3 has been suggested to allow T_regs_ to co-localize with CXCR3+ T_H_1 effector T cells at inflammatory sites^6,7^. We thus analyzed whether infection-experienced T_regs_ might show enhanced suppressive capacity in a localized re-challenge model of infection. Injection of LCMV into the footpad of mice induces a tissue localized anti-viral immune response that results in effector T cell influx and tissue swelling^23^. Similarly, epicutaneous VV infection in the ear results in a localized infection with CXCR3-dependent and infection site-restricted recruitment of virus-specific CD8+ T cells^24^. We thus employed these two localized models of infection and tested if infection-experienced T_regs_ could migrate more efficiently to sites of ongoing T_H_1 mediated inflammation and if they could reduce the local effector response (as measured by tissue swelling) at the infected site. We also assessed the endogenous effector T cell responses to address the possibility of a potentiated immune regulation exerted by infection-experienced T_regs_.

**Figure 4:**
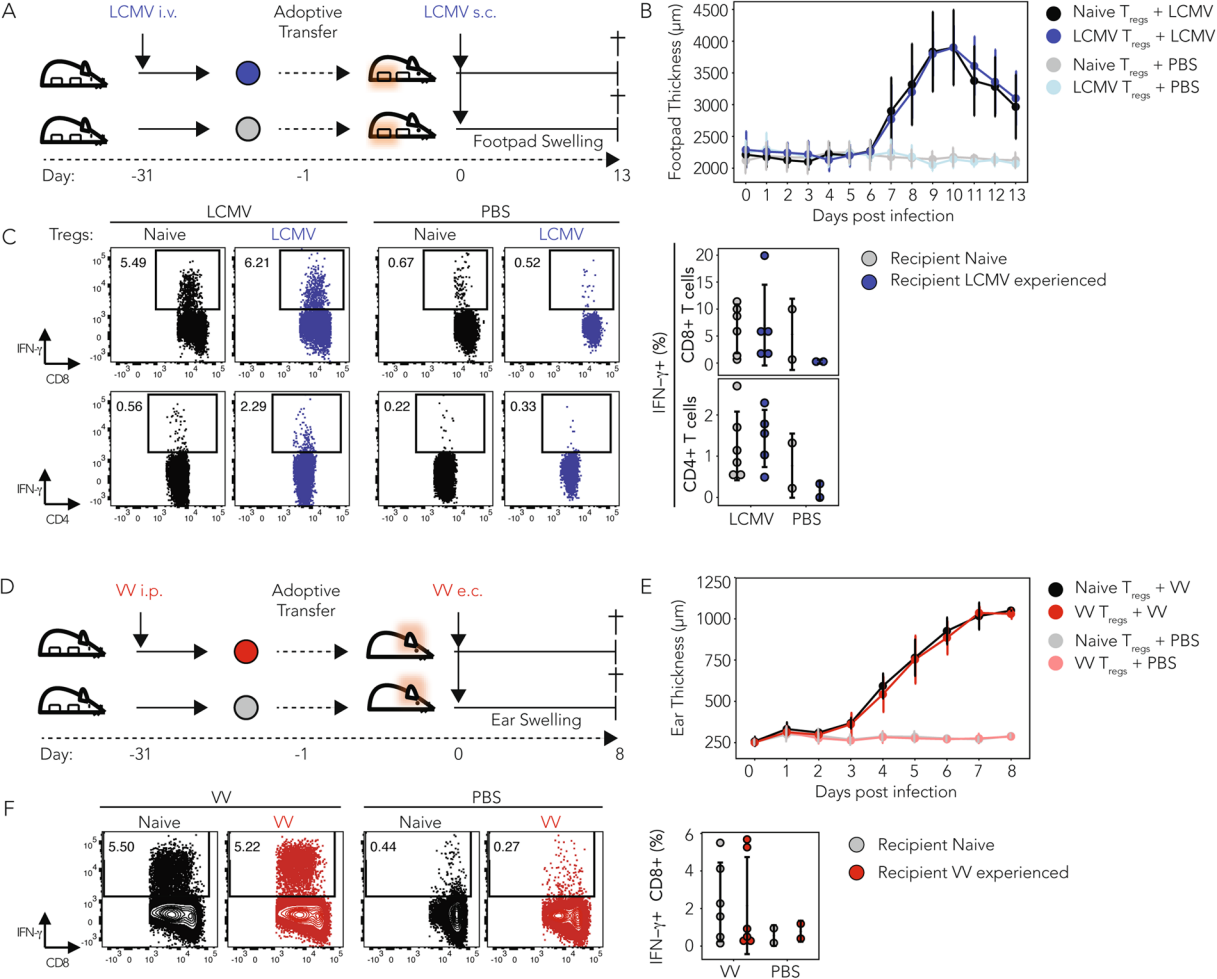
Adoptive transfer of infection experienced T_regs_ does not alter tissue swelling and effector T cell responses in localized, homologous viral challenges. CD4+GFP+ T_regs_ were sorted from naive, LCMV-experienced (**A–D**) or VV-experienced (**E–I**) CD45.2+ *Foxp3*-GFP reporter mice 30 days after the primary infection and 500’000 of each population were adoptively transferred into separate groups of CD45.1+ recipient mice. Recipients of naive, LCMV- or VV-experienced T_regs_ were infected subcutaneously into the hind footpad with LCMV (**A–D**), or epicutaneously into the ears with VV (**E–I**) and the swelling of the infected site was monitored daily for 13 or 8 days, respectively, with a spring loaded caliper. On the final day of the experiment mice were sacrificed and T cell responses in the site draining popliteal lymph node (**A–D**), or cervical lymph node (**E–I**) characterized by FACS. (**A**) Schematic outline of the homologous LCMV challenge experiment. (**B**) Thickness of the footpads of recipients of naive or LCMV-experienced T_regs_ infected with LCMV in the right hind footpad or mock infected with PBS on the contralateral side. (**C**) Cells from the footpad draining popliteal lymph node of recipient mice of the PBS treated side were pooled and from the infected side treated individually and re-stimulated with LCMV peptides (LCMV gp61 and gp33, 4 h) followed by intracellular cytokine staining for IFN-γ. Representative plots (left) and summary graphs (right) are shown. (Mean ± SD; biological replicates: naive n = 6, LCMV-experienced n = 5; 2 independent experiments) (**D**) Schematic outline of the experimental setup to elicit homologous, localized immune challenges using VV in the ear. (**E**) Thickness of the ears of recipients of naive or VV-experienced T_regs_ that were either infected with VV epicutaneously or mock injected with PBS. (**F**) Cells from the ear draining cervical lymph node of recipient mice of the PBS treated side were pooled and from the infected side treated individually and re-stimulated with VV peptide (4 h) followed by intracellular cytokine staining for IFN-γ. Representative plots (left) and summary graphs (right) are shown. (Mean ± SD; biological replicates: naive n = 6, VV-experienced n = 6; 2 independent experiments) (t Test*p < 0.05, **p < 0.01, ***p < 0.001).

In the LCMV model, we performed subcutaneous infections in the hind footpad of mice that had received either LCMV-experienced or naive T_regs_ (Fig. 4A). The swelling of the infected site was measured daily and mice were sacrificed 13 days after local LCMV re-challenge. We did not observe differences in the swelling of the footpads between recipients of naive or LCMV-experienced T_regs_ (Fig. 4B), nor did we observe differences in the frequencies of IFN-γ+ CD8+ or CD4+ effector T cells in the footpad draining popliteal lymph node after virus-specific or unspecific re-stimulation (Fig. 4C and Supplementary Fig. 5A). In line with these results, recipients of naive or VV-experienced T_regs_ did not show differences in response to epicutaneous VV re-challenge, neither in ear swelling (Fig. 4D,E), nor in the frequency of IFN-γ producing CD8+ effector T cells in the ear draining cervical lymph node (Fig. 4F), nor upon stimulation with PMA/Ionomycin (Supplementary Fig. 5B). Furthermore, neither naive nor infection experienced T_regs_ could be detected in the footpad draining or ear draining lymph node after local LCMV or VV infection, respectively. In summary, we could not observe differences in the recruitment or suppressive function of infection experienced and naive T_regs_.

## Discussion

In this study, we investigated the phenotypic and functional features of polyclonal, infection-experienced T_regs_ *in vitro*, and in different T_H_1 responses *in vivo*. Using adoptive (co-)transfer experiments we found that neither the maintenance nor the suppressive function of infection-experienced T_regs_ was altered *in vivo*. Homologous, systemic viral re-challenges using LCMV or VV revealed that infection-experienced T_regs_ did not display an altered composition, activation, nor differences in cell cycling. Furthermore, infection-experienced polyclonal T_regs_ were unable to significantly dampen systemic or localized effector T cell responses compared to T_regs_ isolated from naive mice. Overall these results suggest that T_regs_, which have experienced a viral infection do not show an altered phenotype nor function compared to naive T_regs_ and thus do not display characteristics of a memory response.

Addressing the possibility that T_regs_ generate immunological memory is uniquely challenging and conflicting evidence for and against it has been reported. Several studies employed TCR transgenic T_regs_ and presented compelling evidence that pathogen-specific T_regs_ can generate memory cells. In an Influenza A virus (IAV) infection model, virus-specific T_regs_ underwent pronounced proliferation and contraction phases and yielded a small population of virus-specific T_regs_ that persisted long-term and, upon antigen-specific re-challenge, were recruited more effectively to the lung, expanded more rapidly and potently suppressed effector T cell expansion and cytokine production^16^. Very similar experiments were performed by another group that also reported enhanced production of IL-10 by influenza virus-specific T_regs_ upon antigen-specific re-challenge^15^. In our study, we went beyond the limitations set by models that use TCR transgenic cells and performed adoptive transfers of polyclonal T_regs_ from naive or infection-experienced donor mice. Interestingly, we found no evidence of T_reg_ memory generation or more potent regulation of effector T cell responses.

Aside from the potentially enhanced functionality of memory T_regs_, other researchers have addressed their ability to migrate to peripheral sites and have shown that T_regs_ can form tissue resident memory cells^22^. A set of studies investigated responses of TCR transgenic T_regs_ in a model of inducible antigen expression in keratinocytes^25^ and found robust activation, proliferation and migration of antigen specific T_regs_ to the inflamed skin, reported persistence of a tissue resident, antigen-specific T_reg_ population in the absence of antigen expression and an accelerated response kinetic and disease amelioration upon local antigen re-expression^22^. The fact that in our systemic and local viral re-challenges we could not observe enhanced responses by infectionexperienced T_regs_ may be due to the fact that systemic viral infections, unlike tissue localized infections, may not be suited to generate T_reg_ memory. It is possible that memory T_regs_ are primarily generated in peripheral tissues and persist as tissue resident memory cells, in which case we would not be able to detect them in our experimental models. Nevertheless, based on the reported sustained expression of T-bet^5,6^ and pronounced re-expansion of TCR transgenic memory T_regs_ *in vivo*^15,16,22^ one could suspect an enhanced capacity for homing of memory T_regs_ to sites of local T_H_1 mediated inflammation. However, we did not find evidence for enhanced T_reg_ homing in local re-challenges. In addition, we were unable to recover any infection-experienced T_regs_ from the lymph nodes that drained the site of infection. While our experimental system allows for the analysis of a polyclonal T_reg_ response, we could not observe any expansion of T_regs_ specific for the immunodominant LCMV effector T helper epitope gp66 *in vivo*. This is in contrast to previous reports, where HA-specific T_regs_ were found to expand during antigen-specific primary and secondary challenge i*n vivo*^15^. However, these observations were made in a TCR transgenic model system, where TCR transgenic T_regs_ receive very strong TCR stimulation. It is possible that the LCMV-specific T_regs_ we detected in naive mice receive weaker TCR signals *in vivo*, possibly because they represent self-specific T_regs_ with weak cross-reactivity for LCMV^26^. T_reg_-mediated immune regulation has been implicated in playing a role in various viral infections such as Friend Virus^27^, Cytomegalovirus^28^, hepatitis C virus^29^, or influenza virus infections^30^. Interestingly, influenza virus-specific T_regs_ have been found to potently regulate anti-viral effector T cells responses in influenza infection *in vivo* and show enhanced suppression upon re-challenge^16^. However, it remains to be elucidated whether virus-specific T_regs_ are also generated against other viruses. Our results suggest that LCMV-specific T_regs_ do not undergo expansion upon LCMV infection *in vivo*, possibly due to low affinity for LCMV antigens, which may have precluded memory generation. Indeed, despite clear evidence for the immunoregulatory functions of T_regs_, T_reg_ memory generation is absent in infections with, for instance, Friend Virus^27^, MCMV^28^, or HSV^31^ for which pathogen-specific T_regs_ have not been described so far. Thus, despite earlier reports of murine T_regs_ specific for various pathogens including LCMV^11–14^ it still remains to be established if such cells do get activated in a viral infection and can generate functional memory. Similarly, several studies also presented evidence that argued against T_reg_ memory. One recent study reported that activation-induced changes in gene expression by polyclonal T_regs_ in a self-resolving T_H_1 polarized autoimmune disease were either fully or significantly reversed within 60 days of onset and resolution of the disease^32^. This suggested that no functional T_reg_ memory was formed in a systemic T_H_1 polarized immune response and our data would suggest a similar conclusion. However, another study by the same group employed an intricate T-bet dual reporter system and showed that polyclonal T_regs_ that had expressed T-bet at the peak of a primary immune response directed against *Listeria monocytogenes* expanded more robustly than T_regs_ that *de novo* upregulated T-bet in a secondary, homologous re-challenge with the same pathogen^6^. This observation suggested that infection-experienced T-bet+ T_regs_ are maintained long-term and retain a capacity for enhanced proliferation upon homologous re-challenge. Our data does, however, not confirm these findings as we did not observe increased absolute numbers, nor frequencies of T-bet+ , CXCR3+ or Ki67+ cells among infection-experienced T_regs_ during homeostasis in donor animals nor during homologous systemic or localized re-challenge. As such, our study would argue against LCMV or VV pathogen-specific T_reg_ memory but it will be important to confirm this finding in other models that allow for the analysis of polyclonal, non-TCR transgenic T_reg_ populations.

## Methods

### Mice, pathogens, and infections

C57BL/6 (B6) mice were purchased from Janvier Laboratories and *Foxp3*-GFP.KI reporter^33^ and SMARTA^34^ mice have been described previously. All mice were housed and bred in SPF or OHB facilities at LASC Zurich, Switzerland. All experiments were reviewed and approved by the cantonal veterinary office of Zurich and were performed in accordance with Swiss legislation.

Lymphocytic choriomeningitis virus (LCMV) WE was propagated on L929 fibroblast cells, Vaccinia Virus (VV) on BSC40 cells. Sex- and age-matched mice, 6–12 weeks of age were infected i.v. with 200 ffu LCMV, or i.p. with 2 × 10^6^ ffu VV. For footpad infections 50 μL of PBS containing 500 ffu of LCMV were injected s.c. into the right hind footpad^23^. Epicutaneous ear infections were modified from a previous report^24^ and performed by piercing the ear five times with a bifurcated needle that was dipped into VV stock (7.65 × 10^8^ ffu/mL) in between punches. The contralateral footpads or ears served as a control and were mock infected in an identical manner using sterile PBS. Swelling of the infected site was monitored daily with a spring-loaded caliper.

### Isolation of leukocytes

Harvested spleens or lymph nodes were dissociated by carefully pushing them through a fine metal mesh or 70 μm cell strainer, respectively, followed by red blood cell lysis using ACK buffer (155 mM NH_4_Cl, 1 mM KHCO_3_, 0.1 mM NA_2_EDTA in ddH_2_O, pH 7.2–7.4) for spleen samples, prior to staining for flow cytometry. Bone marrow was flushed out from the femur and tibia-fibula. For isolation of lung cells, mice were perfused with sterile PBS, lungs resected and digested in RPMI medium containing DNase I (200 μg/ml, VWR) and Collagenase I (2.4 mg/ml, Gibco), and dissociated using a gentleMACS (Miltenyi) according to the manufacturer’s protocol. Leukocytes were purified over a 30% Percoll (GE Healthcare) gradient and then stained for flow cytometry.

### Flow cytometry

Single cell suspensions from the indicated organs were surface stained with fluorophore-conjugated antibodies for 20 min, followed by fixation/permeabilization for 45 min using the Foxp3 Staining Buffer Set (eBioscience) for transcription factor staining or 10 min using BD Fixation/Permeabilization Solution kit (BD Bioscience) for intracellular cytokine staining, followed by intracellular staining for 40 min at room temperature. For gp66 tetramer staining, cells were pre-incubated with APC-conjugated tetramer for 20 min before surface staining for additional markers. For intracellular cytokine staining, isolated bulk splenocytes were re-stimulated using phorbol 12-myristate 13-acetate (PMA, 50 ng/mL) and Ionomycin (1 μg/ml), αCD3 (2 μg/ml, BioXcell), the immunodominant LCMV peptides gp61 and gp33 (1 mg/ml; gp61: GLKGPDIYKGVYQFKSVEFD; gp33-41: KAVYNFATM) or the immuno-dominant VV CD8+ T cell epitope (10 mM, TSYKFESV, GenScript) for 4 h at 37 °C in the presence of Brefeldin A (5 μg/mL, BioLegend). Fluorophore-conjugated antibodies against murine CD4 (RM4–5 or GK1.5), CD8 (53–6.7), Foxp3 (FJK-16s), T-bet (4B10), CXCR3 (CXCR3-173), CD44 (IM7), CD85k (H1.1), TIGIT (1G9), CD25 (PC61), IFN-γ (XMG1.2), TNF-α (MP6-XT22) or Ki-67 (16A8) were purchased from BioLegend, eBioscience, or R&D Systems. The LIVE/DEAD Fixable Near-IR Dead Cell Stain Kit (Invitrogen) was used to exclude dead cells. Counting beads (CountBright, Invitrogen) were added before flow cytometric acquisition to determine absolute cell numbers. Data were acquired on a BD LSRFortessa or BD FACSCanto II cytometer (BD Biosciences). Data were analyzed using Flowjo Software (Flowjo, LLC) and where necessary target populations were concatenated to simplify analysis.

### Cell sorting by FACS

Total CD4+ T cells from virus-infected or naive control *Foxp3*-GFP.KI mice were isolated from bulk splenocytes and peripheral lymph nodes (inguinal, brachial, axillary, cervical) using anti-CD4 positive or negative selection beads (BioLegend), followed by FACS sorting. T_regs_ were FACS sorted as CD4 + GFP + cells from *Foxp3*-GFP reporter mice. Cell sorting was performed on a FACSAria III (BD Biosciences).

### Adoptive cell transfers

5 × 10^5^ sorted CD4 + GFP + T_regs_ from infection-experienced (day > 30) or naive *Foxp3*-GFP reporter mice suspended in 200 μl PBS were adoptively transferred by i.v. injection into congenically marked naive recipient mice. The *in vivo* maintenance of the transferred T_reg_ cells was assessed 21–28 days post transfer in the indicated organs. For systemic and localized homologous challenges the T_reg_ recipient mice were infected with LCMV or VV one day after the adoptive transfer.

### T_reg_*in vitro* suppression assays

Suppression assays were performed as described before^8^. Briefly, cells were cultured in DMEM medium supplemented with 10% heat-inactivated FCS, β-mercaptoethanol (50 mM), sodium pyruvate (1 mM, Gibco), non-essential amino acids (Gibco), MEM vitamins (Gibco), penicillin (50 U/mL, Gibco), streptomycin (50 µg/mL, Gibco), and Glutamine (2 mM, Gibco). T_H_1 effector cells were isolated by FACS from spleen and lymph nodes of day 10–14 LCMV-WE infected *Foxp3*-GFP reporter mice as CD4+GFP− cells. CD4+GFP− effector cells (4 × 10^4^ cells/well) and CD4+GFP+ T_reg_ cells were co-cultured in triplicate in the presence of soluble anti-CD3 (1 µg/ml, BioXcell) and irradiated splenic APCs (2 × 10^5^/well) to provide co-stimulation at 37 °C and 5% CO2. After 48 h cells were pulsed with 1 µCi [^3^H]thymidine (PerkinElmer) for an additional 18–22 h, then harvested and [^3^H]thymidine incorporation was assessed to analyze T cell proliferation. Percent suppression = ([mean value of C.P.M. of wells with CD4+Foxp3− cells alone - C.P.M. of well with the indicated ratio of effector T: T_reg_ cells]/[mean C.P.M. of wells with CD4+Foxp3− effectors alone])*100. Cytokine secretion was determined in supernatants harvested after 48 h of co-culture using the LEGENDplex Assay according to the manufacturer’s instructions (BioLegend).

### Statistical analysis

Statistical significance between two individual groups was determined using two-sided t Test, for more than two groups one-way ANOVA was used. Assessment was performed using Prism7 or R software. Statistical significance values are indicated as follows: p < 0.05 (*), p < 0.01 (**) and p < 0.001 (***).

### Ethical approval and informed consent

All animal experiments were reviewed and approved by the cantonal veterinary office of Zurich and were performed in accordance with Swiss legislation as detailed in the methods section.

## Supplementary information

Supplementary information.

## Data Availability

All relevant data of the article and its supplementary files are available from the corresponding author upon request.
