# Supplementary material for: Preceding Viral Infections Do Not Imprint Long-Term Changes in Regulatory T Cell Function
Source: Sci Rep. 2020 May 20;10:8350. doi: 10.1038/s41598-020-65212-9 (PMC7239864; doi:10.1038/s41598-020-65212-9)
Supplement: Supplementary file 1 — Supplementary information. [file 41598_2020_65212_MOESM1_ESM.pdf]

SUPPLEMENTARY INFORMATION

Preceding Viral Infections Do Not Imprint Long-Term Changes in Regulatory T Cell Function

Felix Rost[1], Katharina Lambert[1,2], Nikolas Rakebrandt[1,3], Nicole Joller[1*]

[1]University of Zurich, Institute of Experimental Immunology, Zurich, 8057, Switzerland

[2]current address: Translational Research Program, Benaroya Research Institute, Seattle, WA, 98101, USA

[3]current address: F.Hoffmann-La Roche, Basel, 4070, Switzerland

[*] Corresponding author: nicole.joller@immunology.uzh.ch

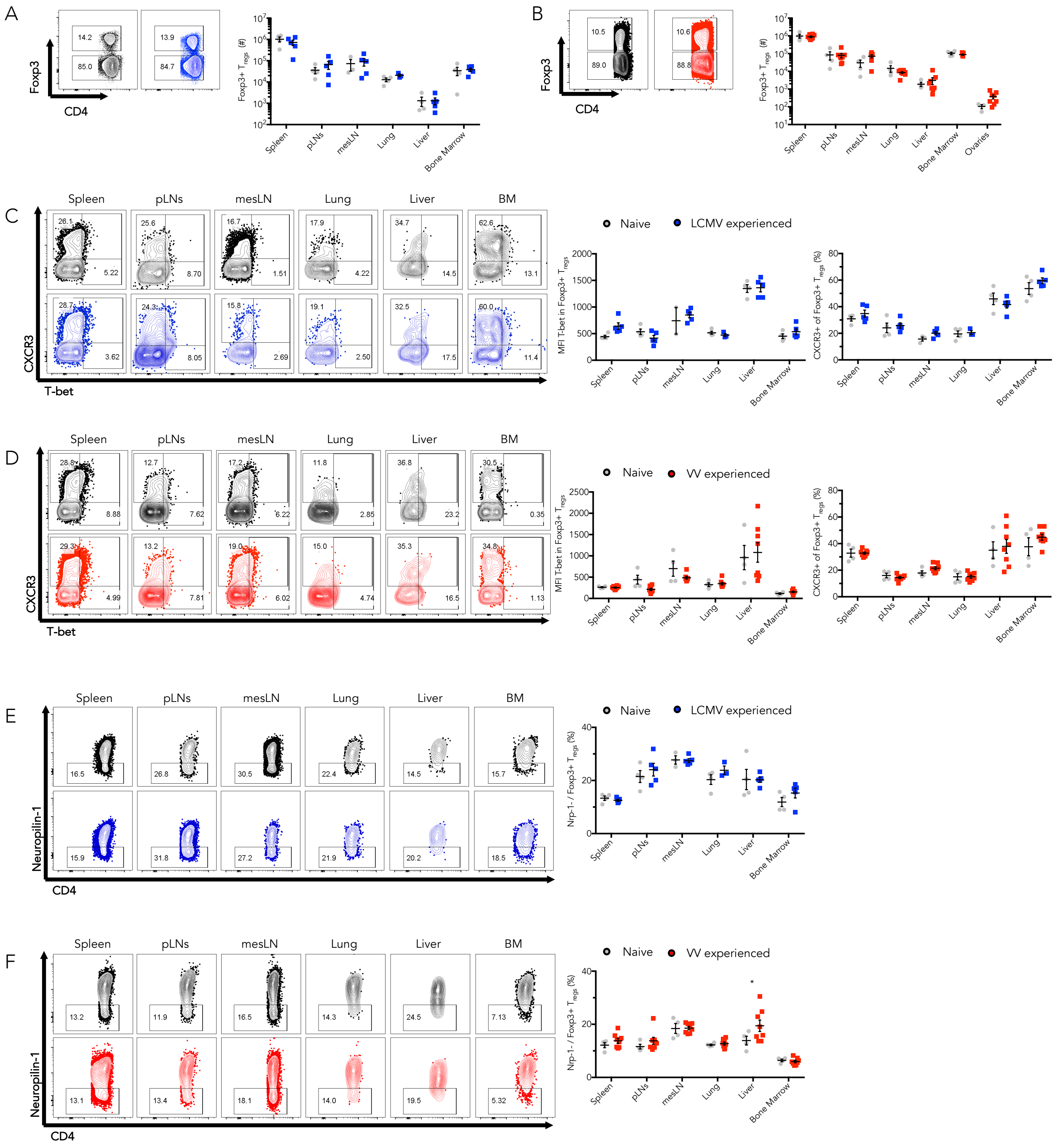

Supplementary Figure 1: T_{reg} profiling in peripheral organs of naive, LCMV or VV infection experienced mice. C57BL/6 mice were acutely infected with LCMV (blue) or VV (red) or left uninfected (grey) and sacrificed 30-60 days post infection. Representative plots (left) and absolute numbers (right) of CD4+ Foxp3+ T_{regs} (A,B), T-bet MFI and frequency of CXCR3+ T_{regs} (C,D) and frequencies of Neuropilin-1- pT_{regs} (E,F) in LCMV, VV experienced mice and naive controls as determined by flow cytometry in the indicated organs (Mean ± SD; biological replicates: naive LCMV / VV = 4 / 4, infection experienced LCMV / VV = 5 / 8; 1-2 independent experiments) (two way ANOVA followed by Sidak's multiple comparisons test: *$p < 0.05$).

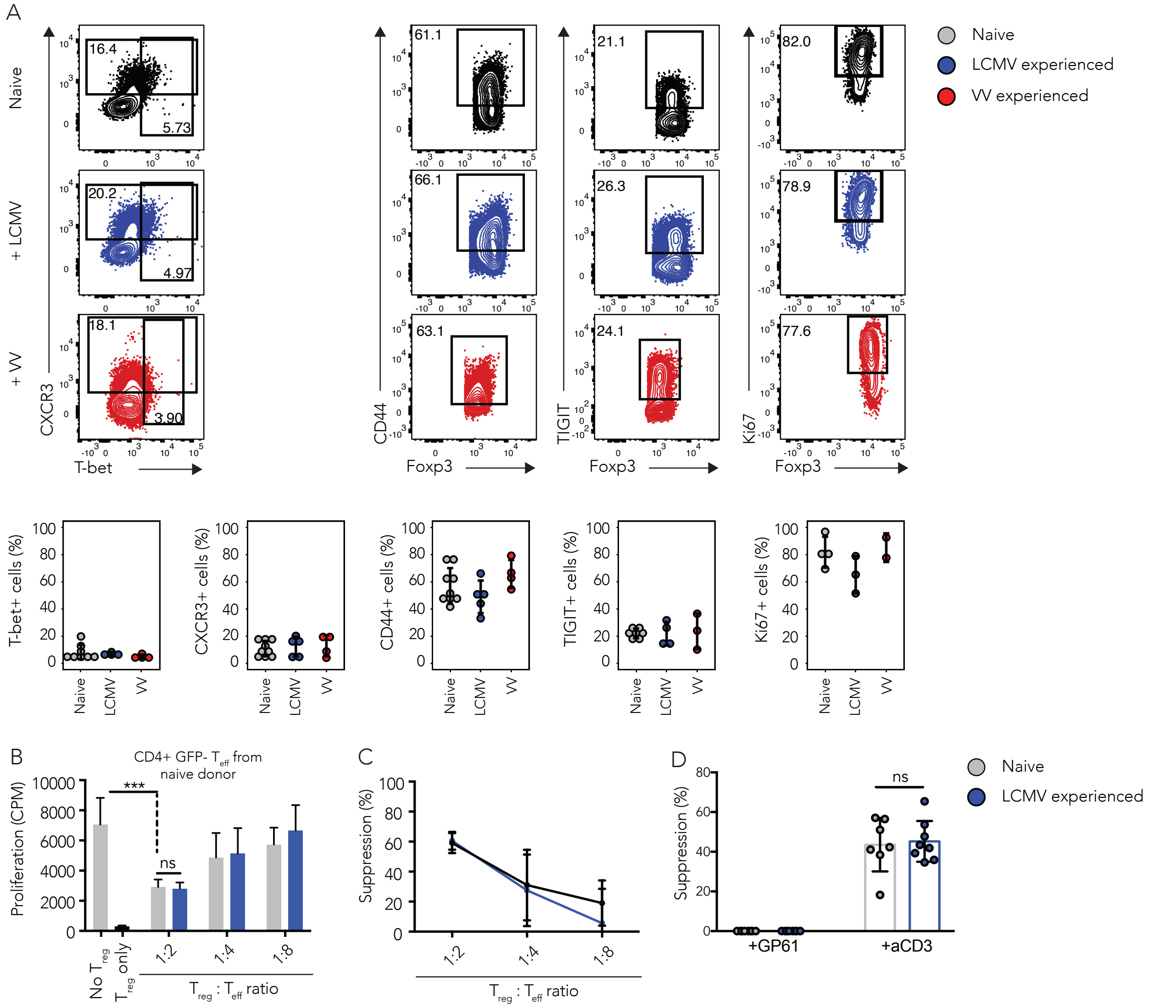

Supplementary Figure 2: Isolated infection experienced and naïve T_{reg} share similar phenotypes (A) $CD4^{+}GFP^{+}$ T_{regs} were sorted from spleen and pooled peripheral lymph nodes of LCMV experienced or VV experienced *Foxp3*-GFP reporter mice 30 days post infection or naive control mice and their phenotype was analyzed by flow cytometry. A) Frequency of T-bet+, CXCR3+, CD44+, TIGIT+ or Ki67+ of sorted T_{regs} before adoptive cell transfer. Representative (top) and summary plots (bottom) are depicted. (Mean ± SD; biological replicates: naive n = 4-8, LCMV experienced n = 3-5, VV experienced n= 2-4; 2-8 independent experiments) (B) $CD4^{+}GFP^{+}$ T_{reg} were sorted from naive or LCMV experienced *Foxp3*-GFP reporter mice and co-cultured with CD4+ GFP- effector cells isolated from naive *Foxp3*-GFP reporter (B,C) or LCMV gp61-specific SMARTA mice (D) in the presence of soluble anti-CD3 (1 µg/ml) (B,C,D) or gp61 (1ug/mL) (D) and irradiated splenic APCs isolated from naive mice for 2 days, then $[^{3}H]$thymidine was added for 18-22h, and $[^{3}H]$thymidine incorporation was quantified to determine proliferation. Target cell proliferation (B) and calculated T_{reg} mediated suppression (C, D) are depicted. (Mean ± SD; biological replicates: B, C: no T_{regs}, naive T_{regs}, LCMV experienced T_{regs} n = 6; 2 independent experiments; one way ANOVA and multiple comparisons test, **p* < 0.05, ***p* < 0.01, ****p* < 0.001; D: naive n = 7, LCMV experienced n = 8; 2 independent experiments; t Test)

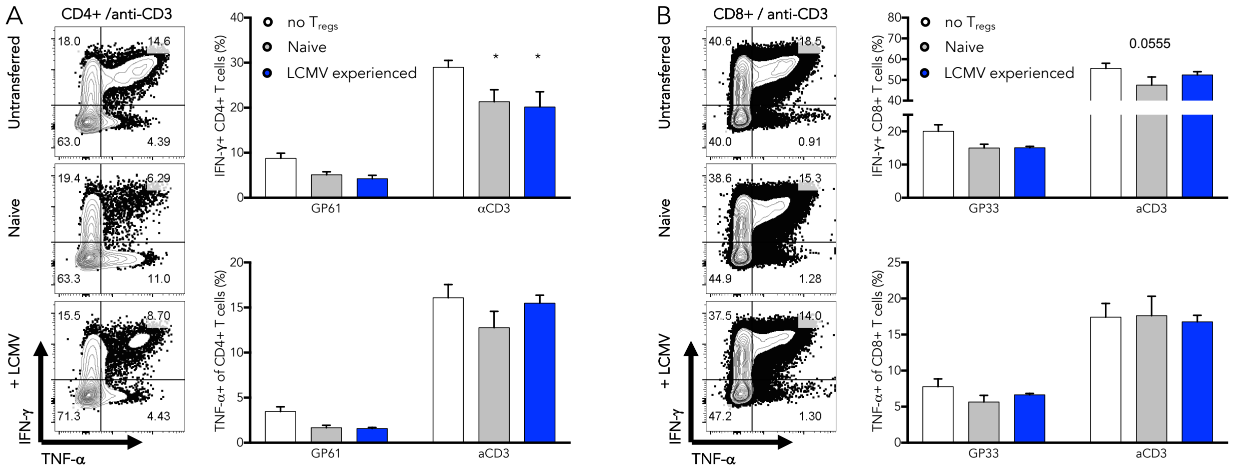

Supplementary Figure 3: Adoptive transfer of 5x10^5 T$_{regs}$ dampens effector T cell responses *in vivo*. CD4+ GFP+ T$_{regs}$ were sorted from naive or LCMV experienced CD45.1+ *Foxp3*-GFP reporter mice 30 days after the primary infection and 500'000 of each population were adoptively transferred into separate groups of naive, CD45.2+ recipient mice followed by acute LCMV infection. A separate group of mice that did not receive T$_{regs}$ was also infected with LCMV and served as a control. Mice were sacrificed 10 days post LCMV infection and spleens were harvested to address the endogenous CD4+ (A) and CD8+ (B) effector T cell response. Splenocytes were re-stimulated with gp61 and gp33 or anti-CD3 for 4 h or 3 h, respectively, followed by intracellular cytokine staining for IFN-γ and TNF-α. (Mean ± SD; biological replicates: no T$_{regs}$ = 6, naive n = 6, LCMV experienced n = 4; 2 independent experiments) (t Test: *p < 0.05).

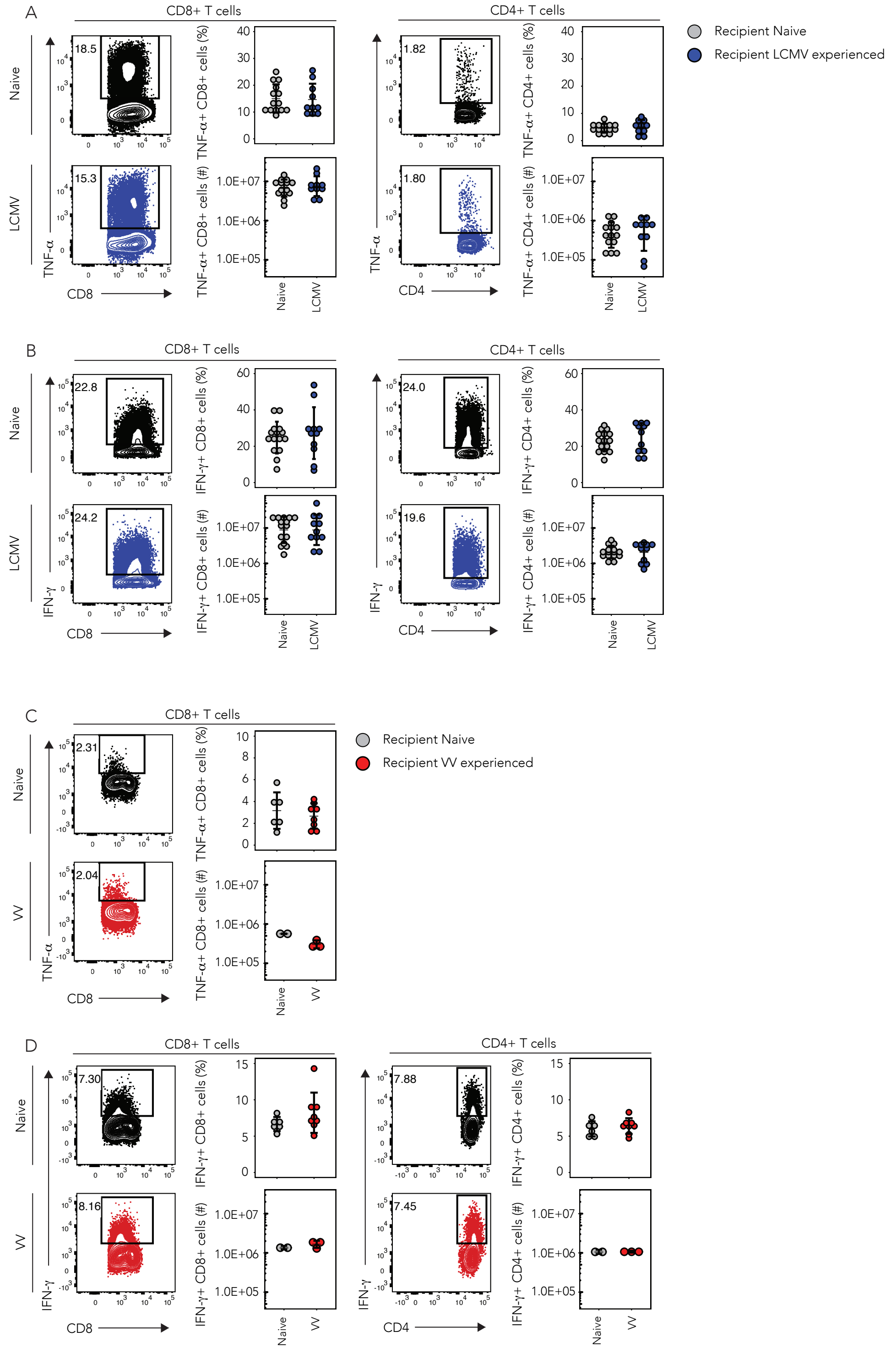

Supplementary Figure 4: Recipients of naive or infection experienced T_{regs} show comparable effector T cell responses in systemic, homologous re-challenges. $CD4^{+}GFP^{+}$ T_{regs} were sorted from naive, LCMV (A-B) or VV (C-D) experienced CD45.1+ *Foxp3*-GFP reporter mice 30 days after the primary infection and 500'000 of each population were adoptively transferred into separate groups of CD45.2+ recipient mice followed by acute LCMV (A-B) or VV (C-D) infection one day later. T_{reg} recipients were sacrificed 10 or 7 days after LCMV or VV re-challenge, respectively. Cells from the spleen of recipient mice were re-stimulated *ex vivo* with LCMV peptides (LCMV gp61 and gp33) (A), PMA-Ionomycin (B, D) or with VV peptide (C) for 4 h in the presence of Brefeldin A, followed by intracellular cytokine staining for TNF-α (A, C) or IFN-γ (B, D) Representative plots (left) and summary graphs for frequencies and absolute numbers of cytokine producing cells (right) are depicted. Cumulative data from 2-4 independent experiments are shown (Mean ± SD; biological replicates: LCMV: naive n = 14, infection experienced n= 11; 3 independent experiments; VV: naive n = 6, infection experienced n= 2-6; 2 independent experiments) (t Test, *$p < 0.05$, **$p < 0.01$, ***$p < 0.001$).

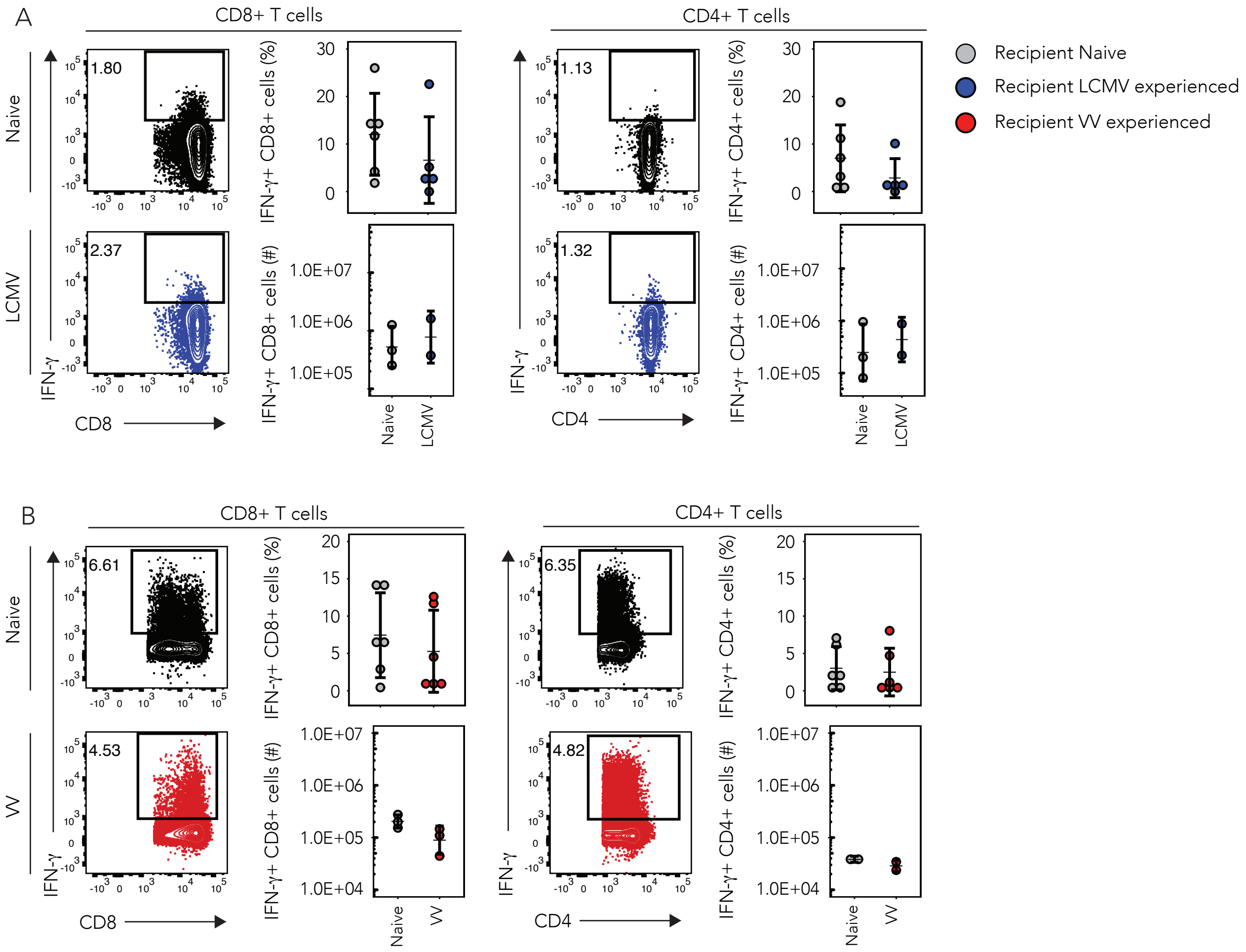

Supplementary Figure 5: Recipients of naive or infection experienced T$_{regs}$ show comparable effector T cell responses in localized, homologous re-challenges. CD4$^+$GFP$^+$ T$_{regs}$ were sorted from naïve, LCMV (A) or VV (B) experienced CD45.1+ *Foxp3*-GFP reporter mice 30 days after the primary infection and 500’000 of each population were adoptively transferred into separate groups of CD45.2+ recipient mice followed by acute, subcutaneous LCMV infection in the right hind footpad (A) or epicutaneous VV infection in the right ear (B) one day later. T$_{reg}$ recipients were sacrificed 13 or 8 days after LCMV or VV re-challenge, respectively. Cells from the footpad draining popliteal lymph node (A) or the ear draining cervical lymph node (B) of recipient mice were re-stimulated *ex vivo* with PMA-Ionomycin for 4 h in the presence of Brefeldin A, followed by intracellular cytokine staining for IFN-γ. Representative plots (left) and summary graphs for frequencies and absolute numbers of cytokine producing CD8+ (left) or CD4+ T cells (right) are depicted. Cumulative data from 1-2 independent experiments are shown (Mean ± SD; biological replicates: LCMV: naive n = 6, infection experienced n= 5; 2 independent experiments; VV: naive n = 6, infection experienced n= 6; 2 independent experiments) (t Test, *$p < 0.05$, **$p < 0.01$, ***$p < 0.001$).
